# Effects of hydrogen-rich water on aging periodontal tissues in rats

**DOI:** 10.1038/srep05534

**Published:** 2014-07-02

**Authors:** Takaaki Tomofuji, Yuya Kawabata, Kenta Kasuyama, Yasumasa Endo, Toshiki Yoneda, Mayu Yamane, Tetsuji Azuma, Daisuke Ekuni, Manabu Morita

**Affiliations:** 1 Department of Preventive Dentistry, Okayama University Graduate School of Medicine, Dentistry and Pharmaceutical Sciences, 2-5-1 Shikata-cho, Kita-ku, Okayama 700-8558, Japan

## Abstract

Oxidative damage is involved in age-related inflammatory reactions. The anti-oxidative effects of hydrogen-rich water suppress oxidative damage, which may aid in inhibiting age-related inflammatory reactions. We investigated the effects of drinking hydrogen-rich water on aging periodontal tissues in healthy rats. Four-month-old male Fischer 344 rats (n = 12) were divided into two groups: the experimental group (hydrogen-rich water treatment) and the control group (distilled water treatment). The rats consumed hydrogen-rich water or distilled water until 16 months of age. The experimental group exhibited lower periodontal oxidative damage at 16 months of age than the control group. Although protein expression of interleukin-1β did not differ, gene expression of Nod-like receptor protein 3 inflammasomes was activated in periodontal tissues from the experimental group as compared with the control group. Drinking hydrogen-rich water is proposed to have anti-aging effects on periodontal oxidative damage, but not on inflammatory reactions in healthy rats.

Aging is a reality for all living organisms, and is the result of the progressive accumulation of deleterious changes that reduce an organism's ability to resist stress, decreasing the possibility of survival. One of the most popular theories used to explain the mechanisms of aging is the mitochondrial free radical theory of aging^1^,^2^. In this theory, mitochondrial function declines in an age-dependent manner, thereby enhancing the production of reactive oxygen species (ROS)^3^. Moreover, during aging, it is reported that increased production of ROS induces oxidative mitochondrial DNA (mtDNA) damage, which can stimulate the activation of the Nod-like receptor protein (NLRP) 3 inflammasome in tissues^4^. Because NLRP3 inflammasomes serve as a platform for caspase-1 activation and subsequent proteolytic maturation of the potent pro-inflammatory cytokine interleukin (IL)-1β^4^,^5^, these indicate that oxidative mtDNA damage can stimulate age-related inflammatory reactions in tissues. Therefore, reduction in oxidative mtDNA damage through antioxidant therapy may be effective in suppressing age-related inflammatory reactions.

Molecular hydrogen is considered a novel antioxidant that can reduce oxidative damage^6^. Drinking hydrogen-rich water is an alternative mode of molecular hydrogen delivery. Clinical and animal studies have demonstrated that drinking hydrogen-rich water provides numerous health benefits, such as in regards to the serum lipid profiles of those at risk for metabolic syndrome^7^,^8^, quality of life after radiation exposure^9^, oxidative damage in patients with chronic hepatitis B^10^ and nonalcoholic steatohepatitis related hepatocarcinogenesis^11^. In a previous study, we also found that drinking hydrogen-rich water suppressed ligature-induced periodontal inflammation in a rat model^12^. Notably, these studies mainly focused on the effects of hydrogen-rich water on human disease status and animal disease models. However, it remains unclear what effects drinking hydrogen-rich water have over the course of a lifetime.

In the present study, we hypothesized that drinking hydrogen-rich water might suppress age-related oxidative mtDNA damage and inflammatory reactions in the periodontal tissue. Thus, the purpose of this study was to investigate the anti-aging effects of drinking hydrogen-rich water on oxidative mtDNA damage and inflammatory reactions in rat periodontal tissue. The main outcomes of this study were oxidative mtDNA damage and IL-1β expression in the periodontal tissue. The level of oxidative damage was determined by measuring 8-hydroxydeoxyguanosine (8-OHdG), which is highly specific for DNA damage^13^. To gain better insight into the mechanism of action of hydrogen-rich water, we analyzed histological changes and NLRP3 inflammasome activation.

## Results

### Body weights and food consumption

Body weights (mean ± SD of 6 rats, g) of the baseline, control and experimental groups were 286 ± 10, 324 ± 28 and 346 ± 16, respectively. There were no significant differences between the control and the experimental groups with regard to food and water consumption, body weight or growth pattern during the experimental period.

### 8-OHdG levels of periodontal tissues and serum

**Figure 1:**
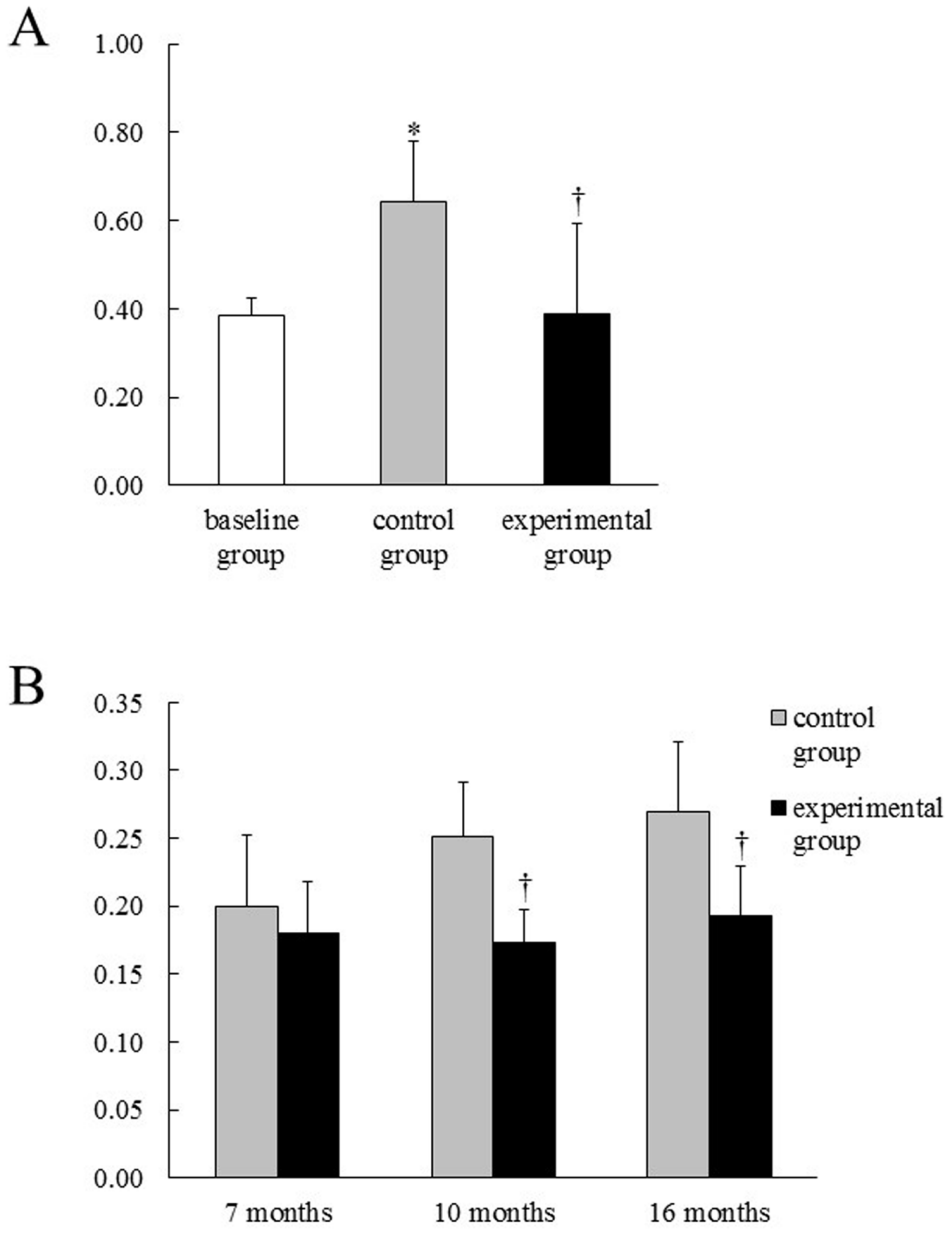
The level of 8-OHdG in rat periodontal tissue (A) and serum (B). Means ± SD of 6 rats. *Significant difference compared to the baseline group. ^†^Significant difference compared to the control group.

Periodontal levels of mitochondrial 8-OHdG were higher in the control group than in the baseline group (p < 0.05), and lower in the experimental group than in the control group (p < 0.05) (Fig. 1A). In the control group, serum levels of 8-OHdG increased in an age-dependent manner (Fig. 1B). In the experimental group, serum levels of 8-OHdG did not change during aging. There were significant differences in serum 8-OHdG levels between the two groups at 10 and 16 months of age.

### Histopathological analysis

**Figure 2:**
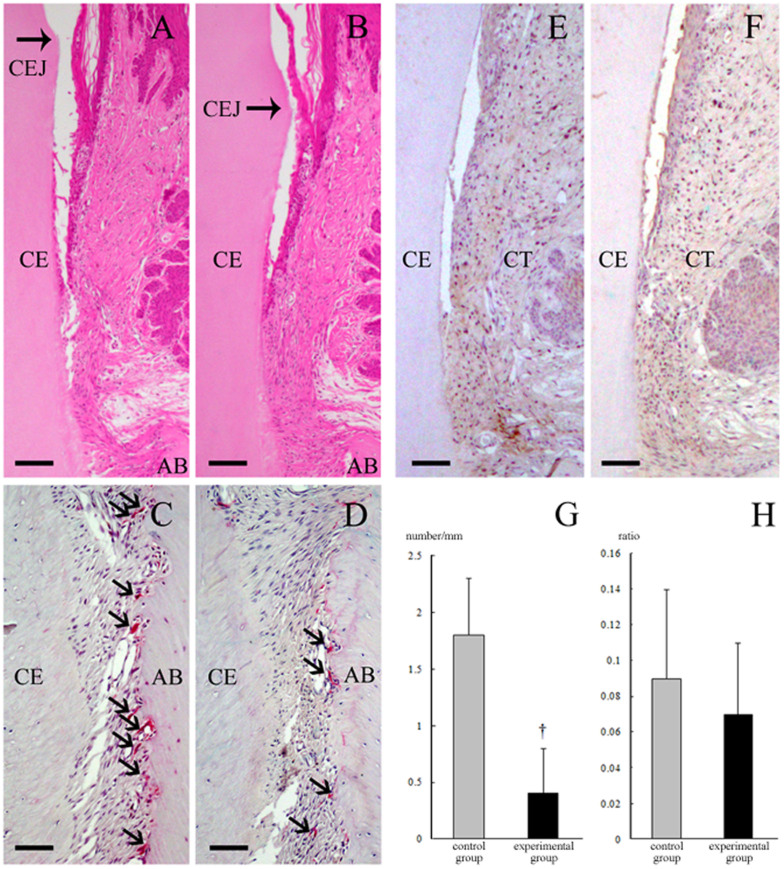
Photomicrographs of rat periodontal tissue. The distance between the cemento-enamel junction and the alveolar bone crest in the control group (A) was greater than that in the experimental group (B). While the control group (C) exhibited greater expression of TRAP-positive osteoclasts (arrows) than the experimental group (D), there was no significant difference in IL-1β expression between the control (E) and experimental groups (F). The number of TRAP-positive osteoclasts (mean ± SD of 6 rats, cell number/mm) was significantly different between the control and experimental groups (1.8 ± 0.5 and 0.4 ± 0.4, respectively; p < 0.05) (G). On the other hand, the ratio of IL-1β positive cells to total cells (mean ± SD of 6 rats) was 0.09 ± 0.05 in the control group and 0.07 ± 0.04 in the experimental group, representing a non-significant difference between the two groups (H). AB, alveolar bone; CE, cementum; and CEJ, cemento-enamel junction; and CT, connective tissue. Bar = 100 μm (A and B) and 40 μm (C–F). In this study, a total of 18 tissue sections (3 from each rat) per group were used in the histological analysis. Thus, the photomicrographs in each group represent 18 independent analyses. ^†^Significant difference compared to the control group.

**Figure 3:**
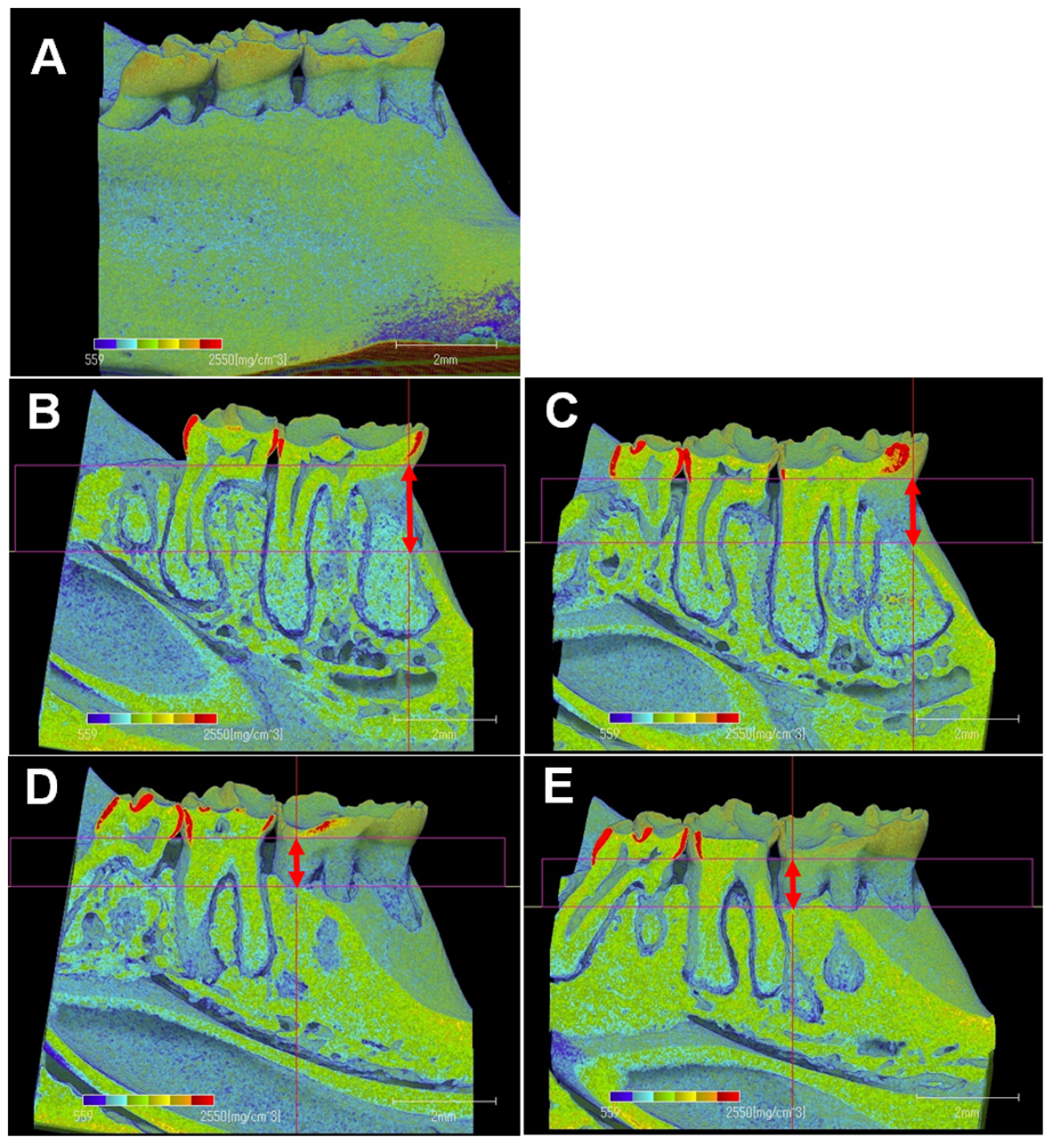
The 3D image model of the mandibular regions in rats (A). In the mesial root regions, the level of alveolar bone loss (arrows) was greater in the control group (B) than in the experimental group (C). In the distal root regions, the level of alveolar bone loss in the control group (D) was similar with that in the experimental group (E).

In the paraffin-embedded bucco-lingual sections, the linear distance between the cemento-enamel junction and the alveolar bone crest (mean ± SD of 6 rats, μm) for the baseline, control and experimental groups was 627 ± 104, 931 ± 45 and 828 ± 45, respectively. The values of the linear distance between the cemento-enamel junction and alveolar bone crest were significantly lower in the experimental group than in the control group (p < 0.05) (Fig. 2A and B). In the 3D image of the first molar, the level of alveolar bone loss for the medial root region, but not the distal root region, was greater in the control group than in the experimental group (Fig. 3). In addition, the numbers of TRAP-positive osteoclasts were lower in the experimental group than in the control group (p < 0.05) (Fig. 2C, D, and G). However, IL-1β protein expression was low in both the control and experimental groups (Fig. 2E and F), and there was no significant difference in the ratio of IL-1β-positive cells to total cells between the two groups (Fig. 2H).

### Bone mineral density of alveolar bone

Bone mineral densities (mean ± SD of 6 rats, mg/cm3) of the control and the experimental groups were 1125 ± 122 and 1175 ± 36, respectively. There was no significant difference between the two groups.

### Gene expression in periodontal tissues

Gene expression of NLRP3, caspase-1, ASC and IL-1β in periodontal tissues obtained from the experimental group was significantly higher in the experimental group than in the control group (p < 0.05) (Table 1). In addition, gene expression of NF-κB was significantly lower in the experimental group than in the control group (p < 0.05).

## Discussion

When hydrogen-rich water was placed into the stomach of rats, molecular hydrogen was detected at the level of several μM in the blood^14^. Molecular hydrogen is known to have anti-oxidative properties^6^. Therefore, drinking hydrogen-rich water may protect against age-related oxidative damage. In this study, the control group showed a higher periodontal level of mitochondrial 8-OHdG than the baseline group. On the other hand, the mitochondrial 8-OHdG level was lower in the experimental group than in the control group. Since 8-OHdG is accepted as a parameter of oxidative DNA damage^13^, the results indicate that hydrogen-rich water could reduce oxidative mtDNA damage in aging periodontal tissues.

Hydrogen-rich water may have the potential to decrease systemic oxidative damage effects in the periodontal tissue. In the control group, the serum level of 8-OHdG increased in an age-dependent manner. In the experimental group, the serum 8-OHdG level did not change with aging, which suggests that the suppressive effect of hydrogen-rich water consumption on age-related oxidative damage was systemic.

In this study, we examined the effects of molecular hydrogen on age-related oxidative damage. However, changes in oxidative damage may be affected besides molecular hydrogen in our model. For instance, molecular oxygen in drinking water has decreased on the process of creating hydrogen-rich water. Therefore, chronic consumption of drinking water with oxygen deprivation may also affect age-related oxidative damage. Further studies are needed to clarify this point.

Oxidative damage is involved in osteoclast differentiation^15^. In our previous study^12^, drinking hydrogen-rich water suppressed osteoclast differentiation following periodontal inflammation. In this study, drinking hydrogen-rich water lowered the number of TRAP-positive osteoclasts on the alveolar bone surface with aging. This indicates that hydrogen-rich water ameliorates the effects of aging on osteoclast differentiation by reducing oxidative damage. In our findings, the distance between the cemento-enamel junction and the alveolar bone crest (the degree of alveolar bone loss) was also lower in the experimental group than in the control group. Drinking hydrogen-rich water is proposed to be effective in suppressing alveolar bone loss as a result of aging and/or periodontal inflammation. On the other hand, there was no significant difference in bone mineral density between the control and the experimental groups. It is feasible that drinking hydrogen-rich water did not induce qualitative change of alveolar bone in our model.

It is reported that the number of osteoclasts on alveolar bone surface decreased from 6 to 100 weeks old^16^. This observation is different from the present observations, which showed the number of TRAP-positive osteoclasts increased from 4 to 16 months old. A previous study using the rats aged 1.5–15 months demonstrates that the number of TRAP-positive osteoclasts in the mesial regions increased with age, while those in the distal regions decreased with age^17^. In this study, we evaluated osteoclastic activities at only mesial regions. Since the osteoclastic activities on alveolar bone surface differ dependent on the anatomical location^18^, the effects of hydrogen-rich water on degree of alveolar bone loss in the distal regions may be different from the present observations.

Studies have shown the antioxidative effects of hydrogen-rich water on various organs. Significantly less superoxide formation in the brain was observed in the hydrogen-rich water consumption group than in the pure water consumption group in vitamin C-depleted SMP30/GNL knockout mice^19^. It is also known that myocardial 8-OHdG concentrations in irradiated mice were significantly lower in the hydrogen-rich water treated groups than in the controls^20^. Moreover, a clinical study demonstrated that drinking hydrogen-rich water decreased serum parameters of oxidative damage and improved liver function in patients with chronic hepatitis B^10^. These findings are consistent with the present results showing that hydrogen-rich water decreased periodontal and serum oxidative damage.

In this study, drinking hydrogen-rich water down-regulated gene expression of NF-κB in the periodontal tissue, which is also involved in IL-1β production^21^. Therefore, we expected that hydrogen-rich water consumption would reduce protein expression of IL-1β; however, IL-1β protein expression was not altered in our model. This discrepancy may be due to other mechanisms. Notably, drinking hydrogen-rich water up-regulated the expression of genes encoding the NLRP3 inflammasome (i.e., NLRP3, ASC, caspase-1 and IL-1β) in this study. In an *in vitro* study, ROS inhibitors blocked priming, but not activation of the NLRP3 inflammasome^22^, which may support that hydrogen-rich water cannot block activation of NLRP3 inflammasomes. On the other hand, activation of NLRP3 inflammasomes is not only mediated by ROS and NF-κB, but could also be indirectly mediated by disturbances in (1) the thiol redox balance between thioredoxin (TRX)/TRX-binding protein, (2) ceramide synthesis, (3) mitochondrial integrity leading to the leakage of mtDNA, and (4) lysosomal stability triggering release of cathepsin B^4^. Further research is required to clarify the underlying mechanisms.

The application of appropriate dietary treatment could prove useful in the management of aging. It was demonstrated that CoQ10 intake for 6 weeks improved age-related impairments in spatial performance of mice^23^. It was also demonstrated that consumption of epigallocatechin gallate reduced kidney damage and improved age-related inflammation and oxidative damage^24^. Furthermore, it is reported that diets based on virgin olive oil or fish oil can prevent age-related alveolar bone loss with induction of biogenesis, autophagy and the antioxidant systems and avoiding mitochondrial electron transport system alterations^25^. In the present study, we observed anti-aging effects of hydrogen-rich water on oxidative damage in the periodontal tissue.

This study has some limitations. First, we did not collect data concerning changes in the populations of microorganisms, as our focus was on the anti-aging effects of hydrogen-rich water on periodontal tissues. In future, it may be necessary to clarify how hydrogen-rich water modulates the etiology of microorganisms during aging. Next, the anti-aging effects of hydrogen-rich water may differ according to the treatment duration. Therefore, the effect of treatment duration on the efficacy of hydrogen-rich water consumption against periodontal aging needs to be determined using the current model.

In conclusion, drinking hydrogen-rich water diminished oxidative mtDNA damage, but did not suppress inflammatory reactions in aging periodontal tissues.

## Methods

### Animals

Four-month-old male Fischer 344 rats (n = 18) were entrained to controlled temperature (23–25°C), 12-h light-dark cycle, and free access to powdered food (MF; Oriental Yeast Co., Ltd., Osaka, Japan) and drinking water. All the animals received humane care in compliance with institutional animal care guidelines, and the experimental protocol was approved by the Animal Research Control Committee of Okayama University (OKU-2012532).

### Experimental design

Six of 18 rats (baseline group) were sacrificed immediately. The remaining 12 4-month-old rats were randomly divided into two groups of six rats each: 1) control group treated with normal water (distilled water), and 2) experimental group treated with hydrogen-rich water. Each type of water was placed in a glass vessel twice daily. The rats consumed distilled water or hydrogen-rich water until reaching 16 months of age. Hydrogen-rich water was prepared by electrolysis of water using the Aquela Legend (AL-036A; Ecomo International Co. Ltd., Fukuoka, Japan)^26^. The principle of water electrolysis is to split water, a compound of hydrogen and oxygen, back into original hydrogen and oxygen. We confirmed that the hydrogen concentration was <1 μg/L in distilled water and >500 μg/L in hydrogen-rich water (1 hour after electrolysis of water), respectively. In a glass vessel, the hydrogen concentration of the hydrogen-rich water was >400 μg/L after 24 hours. In addition, the pH of drinking water did not significantly change by creating the free hydrogen in water.

### Sampling

The animals were sacrificed under general anesthesia with diethyl ether. For histological analysis, the left maxillary molar regions were fixed in 4% paraformaldehyde in 0.1 mol/L phosphate buffer (pH 7.4) overnight and decalcified with 10% tetrasodium-EDTA aqueous solution (pH 7.4) for 4 weeks at 4°C. The mandible molar regions were also fixed in 4% paraformaldehyde in 0.1 mol/L phosphate buffer (pH 7.4) to measure bone mineral dentistry. For measurement of 8-OHdG and real-time polymerase chain reaction (PCR), gingival biopsy samples of the right maxillary molar regions were homogenized using a frozen cell crusher (Microtec Co., Chiba, Japan). For all groups, blood samples were collected from the tail vein of rats at 7 and 10 months of age, and from the heart at 16 months. Serum was separated by centrifugation at 1,500 × *g* for 15 minutes, and stored at −80°C for later analysis.

### Measurement of periodontal and serum 8-OHdG

From the homogenized gingival samples, mtDNA was isolated from rat gingiva using a DNA extractor kit (Wako Pure Chemical Industries, Osaka, Japan)^13^. Isolated mtDNA and serum were analyzed by a competitive enzyme-linked immunosorbent assay method using a “High-sensitive 8-OHdG Check” kit (Japan Institute for the Control of Aging, Shizuoka, Japan). The intra-assay and inter-assay coefficients of variation for this assay were <5%.

### Histopathological analysis

Formalin-fixed tissue samples were embedded in paraffin following dehydration with ethanol and immersion in xylene. The paraffin-embedded bucco-lingual sections (4 μm) from the mesial root area of each first molar were then stained with hematoxylin and eosin or other stains, as described below.

Immunohistochemical staining for IL-1β and tartrate-resistant acid phosphatase (TRAP) staining were carried out. Commercial kits (Nichirei Co., Tokyo, Japan) were used to determine the level of IL-1β. The polyclonal antibody against IL-1β (Santa Cruz, CA, USA) was diluted at 1:200 in phosphate buffered saline^13^. The color was developed with 3-3′-diaminobenzidine tetrahydrochloride. To identify osteoclasts, TRAP activity was detected using the azo dye method^27^. Sections were counterstained with Mayer's hematoxylin.

A blinded single examiner (T. T.) performed the following histometric analyses using a light microscope. The distance between the cemento-enamel junction and the alveolar bone crest (a marker of alveolar bone loss) was measured with a microgrid at a magnification of ×200^28^. The numbers of IL-1β positive cells and total cells in standard areas (0.1 mm^2^ each) adjacent to the cementum (two serial areas from the top of the cementum) were determined^13^. TRAP-positive osteoclasts on the alveolar bone surface were counted using a light microscope at ×400 magnification and reported as number/millimeters^27^. We evaluated intra-examiner reproducibility by double-scoring 10 randomly selected sections at two-week intervals. Intra-examiner agreement with IL-1β positive cells and TRAP-positive osteoclast war greater than 80%.

### Measurement of bone mineral density of the mandibles

**Figure 4:**
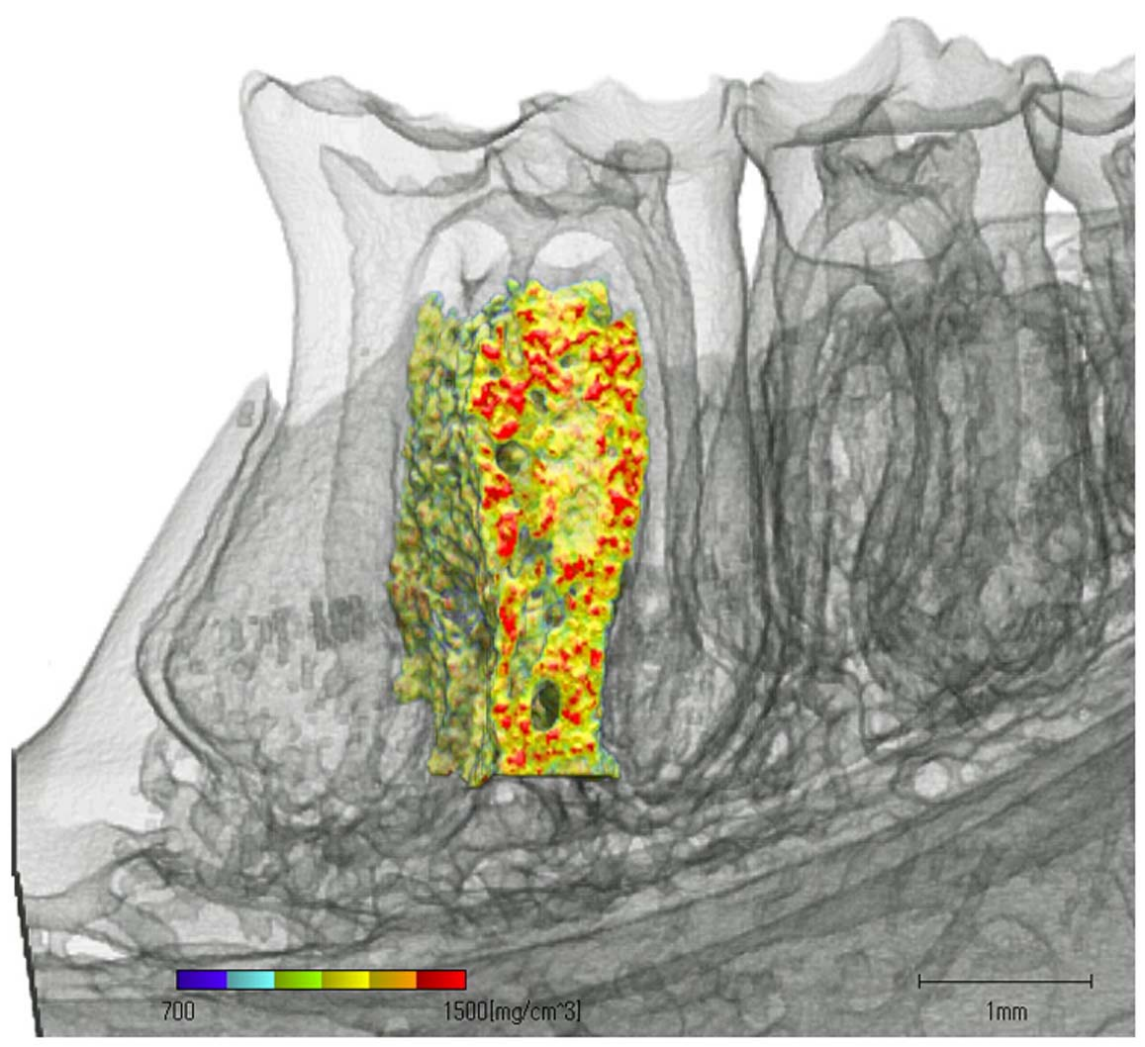
The time base range of bone mineral density of the mandibles. The mandibles from each rat were scanned from the mesial to the distal margin of the first molar. Alveolar bone was defined as the upper part of the mandible, excluding the most apical portion of the root.

The first molar from the mesial to the distal margin were scanned using a computed tomography (TDM1000, Yamato Scientific Co., Ltd, Tokyo, Japan). The computerized tomography was set as follows: voltage, 90 kV; electrical current, 20 μA; 800 projections. After scanning, three-dimensional (3D) models of the mandible were built using a 3D image-analysis system (TRI/3D-BON64, Ratoc System Engineering, Tokyo, Japan) (Fig. 3), and the bone mineral density of the upper part of the mandible, excluding the most apical portion of the root, was calculated (Fig. 4)^29^.

### Real-time reverse transcription PCR

Total RNA was isolated from the gingival biopsy samples using Trizol reagent (Invitrogen, Carlsbad, CA, USA). The purity of the isolated RNA was determined by the 260/280 nm absorbance ratio, and only samples with a ratio of >1.8 were used^30^. Real-time PCR was performed using SYBR Green Realtime PCR Master Mix (Toyobo, Osaka, Japan) in a real-time QPCR system (Agilent Technologies, Tokyo, Japan). The primer sequences of IL-1β, NLRP3, caspase-1, apoptosis-associated speck-like protein (ASC), and nuclear factor (NF)-κB, and β-actin are displayed in Table 2^31^.

Amplification cycling conditions were as follows: 40 cycles at 95°C (30 seconds), 67°C (30 seconds), 72°C (20 seconds) for IL-1β, NLRP3, caspase-1 and ASC; 45 cycles at 95°C (15 seconds), 54°C (20 seconds), 72°C (20 seconds) for NF-κB; 45 cycles at 95°C (10 seconds), 54°C (20 seconds), 72°C (10 seconds) for β-actin. The mRNA levels were calculated in terms of the relative copy number ratio of each mRNA to β-actin for each sample.

### Statistical analysis

The data are expressed as mean values ± standard deviation (SD). Differences in histological data and gingival 8-OHdG levels among the baseline, control, and experimental groups, or time-dependent differences in serum 8-OHdG levels were analyzed using one-way ANOVA followed by Tukey's test. Student's *t*-tests were used for statistical comparisons of gene expression, IL-1β positive ratio, TRAP-positive osteoclasts and 8-OHdG levels between the control and experimental groups. p < 0.05 was considered statistically significant.

## Author Contributions

T.T. conducted most of experiments and analyzed histopathological data. T.T. and D.E. wrote the main manuscript text. Y.K. and Y.E. examined 8-OHdG levels of periodontal tissues and prepared figure1 and table2. K.K. did histopathological analysis and prepared figure2. T.Y. examined gene expression of periodontal tissues and prepared and prepared table1. M.Y. and T.A. assisted some of the experiments. M.M. organized and supervised this study. All authors reviewed the manuscript.

## Supplementary Material

Supplementary Information Nutrient composition of the experimental diet

## Figures and Tables

**Table 1:** Fold Changes of Gene Expression on Rat Periodontal Tissue (N = 6)

	Control group	Experimental group
NLRP3	0.02 ± 0.01	0.16 ± 0.04†
Caspase-1	2.79 ± 0.12	4.86 ± 0.43†
ASC	12.7 ± 28.7	28.7 ± 2.30†
IL-1β	1.70 ± 0.10	6.79 ± 0.36†
NF-κB	36.2 ± 5.96	19.6 ± 9.07†

^†^Significant difference compared to the control group.

**Table 2:** Primer Sequences for NALP3 Inflammasomes

	Sense (5′-3′)	Antisense (5′-3′)	Length (bp)	Accession no.
NALP3	GCTGCTCAGCTCTGACCTCT	AGGTGAGGCTGCAGTTGTCT	165	NM_001191642.1
Caspase-1	TGCCTGGTCTTGTGACTTGGAG	ATGTCCTGGGAAGAGGTAGAAACG	133	NM_172322.1
ASC	TTATGGAAGAGTCTGGAGCTGTGG	AATGAGTGCTTGCCTGTGTTGG	101	NM_012762.2
IL-1β	CACCTCTCAAGCAGAGCACAGA	ACGGGTTCCATGGTGAAGTC	81	NM_031512.2
NFκB	CACTCTCTTTTTGGAGGT	TGGATATAAGGCTTTACG	206	NM_199267.2
β-actin	TGTTGCCCTAGACTTCGAGCA	GGACCCAGGAAGGAAGGCT	155	NM007393
