# Supplementary material for: Effects of hydrogen-rich water on aging periodontal tissues in rats
Source: Sci Rep. 2014 Jul 2;4:5534. doi: 10.1038/srep05534 (PMC4078318; doi:10.1038/srep05534)
Supplement: Supplementary Information — Nutrient composition of the experimental diet [file srep05534-s1.pdf]

Supplementary information

Effects of hydrogen-rich water on aging periodontal tissues in rats

Takaaki Tomofuji, Yuya Kawabata, Kenta Kasuyama, Yasumasa Endo,

Toshiki Yoneda, Mayu Yamane, Tetsuji Azuma, Daisuke Ekuni & Manabu Morita

Table. Nutrient composition of the experimental diet (Oriental MF diet) per 100 g

Nutrient composition	Moisture (g)	7.9
	Crude protein (g)	23.1
	Crude fat (g)	5.1
	Crude ash (g)	5.8
	Crude fiber (g)	2.8
	Nitrogen free extract (g)	55.3
	Calories (kcal)	359
Vitamins	Vitamin A (IU)*	1283
	Vitamin D_3 (IU)	137
	Vitamin E (mg)	9.1
	Vitamin K_3 (mg)†	0.04
	Vitamin B_1 (mg)	2.05
	Vitamin B_2 (mg)	1.1
	Vitamin C (mg)	4
	Vitamin B_6 (mg)	0.87
	Vitamin B_{12} (μg)	5.5
	Inositol (mg)	439
	Biotin (μg)	27
	Panthothenic acid (mg)	2.45
	Niacin (mg)	10.61
	Choline (g)	0.18
	Folic acid (mg)	0.17

3-year average (January 2007-December 2009)

* Retinol

† Amount added

Values are an average of detected lot.

Minerals	Calcium (g)	1.07
	Phosphorous (g)	0.83
	Magnesium (g)	0.24
	Sodium (g)	0.19
	Potassium (g)	0.90
	Iron (mg)	10.6
	Aluminum (mg)	2.1
	Copper (mg)	0.78
	Zinc (mg)	4.89
	Cobalt (mg)	0.10
	Manganese (mg)	4.84
	Ca/P	1.29
	Ca/Mg	4.50
	K/Na	4.77
Amino acids	Isoleucine (g)	0.89
	Leucine (g)	1.74
	Lysin (g)	1.24
	Methionine (g)	0.44
	Cystein (g)	0.36
	Phenylalanine (g)	1.04
	Tyrosine (g)	0.68
	Threonine (g)	0.89
	Tryptophan (g)	0.28
	Valine (g)	1.08
	Arginine (g)	1.42
	Histidine (g)	0.60
	Alanine (g)	1.20
	Aspartic acid (g)	2.14
	Glutamic acid (g)	3.99
	Glycine (g)	1.18
	Proline (g)	1.31
	Serine (g)	1.11
